# Abstract of Proceedings of the Buffalo Medical Association

**Published:** 1865-06

**Authors:** Joseph A. Peters

**Affiliations:** Sec’y.


### ART. II.—*Abstract of Proceedings of the Buffalo Medical Association.*

Tuesday Evening, May 2d, 1865.

Society met pursuant to adjournment, the President, Dr. Ring, in the Chair. Present, Drs. Gould, Rochester, Miner, Strong, Samo, Shaw, Lockwood, Peters, Congar, Gay and Eastman.

The minutes of the last meeting were read and approved.

Dr. George U. Gleason was elected a member upon compliance with the bye-laws, and meanwhile was invited to sit with the Association.

*Dr. Rochester* called attention to a case of empyema, mentioned by him in August last. The patient was a soldier, who had been in service about two years, but for nearly a year was sick in hospital, and was discharged for physical disability. His disease was not recognized, but was considered disease of the heart, liver, etc. He came under the treatment of Dr. Van Pelt of Williamsville, who correctly diagnosticated it and brought the patient to Dr. Rochester. When presented the left thorax was completely filled. There was no resonance except in the supra-scapular and supra-clavicular intercostal spaces. The heart was dislocated upward and to the right, the apex being half an inch to the right of the nipple. There was no evidence of air passing into the lungs. He had chills, night sweats, and dyspnœa; there was great lividity of the lips and nails. Moreover he was of a tubercular family. These cases are considered by Dr. Bowditch very unpromising, nevertheless there was so great suffering that it was resolved to try paracentesis thoracis, and the operation was accordingly performed in the presence of Drs. Eastman, Burwell and Boardman. The instrument used was the one invented by Dr. Wyman and recommended by Dr. Bowditch, which has a pump attached for extracting the air and pus. A small trocar was inserted between the 7th and 8th ribs below the scapula, and by the aid of the pump two quarts of aplastic lymph were evacuated. The patient was then put upon stimulants, and after three days the pump again used and two quarts more obtained. In short, in 3½ months he was tapped twelve times and twenty quarts of purulent matter obtained. The improvement in his physical condition can be best illustrated by stating that he used to come in from Williamsville (10 miles) in the morning, be operated on and return at night. At the conclusion of the treatment his left thorax was almost in normal condition, and the heart was restored to its usual position and healthy. The general treatment consisted of iron, iodide of potassa, cod liver oil and camomile, which has been considered efficacious in arresting suppuration.

Had recently had a similar case which had not so happy a termination. The patient had not been well for a year or two, having had a little cough. He had extreme dyspnœa, lividity of the nails, lips, etc., and cough, but without expectoration. The heart was dislocated to the left side; there was tympanitic resonance over a great portion of the right side, but no respiratory murmur. On forced respiration, amphoric breathing and pectoriloquy were heard. The diagnosis was hydro-pneumo-thorax, with probably perforation of the pleural cavity from tuberculosis. The precise nature of the case was more difficult to determine from the great distress the patient was in, and it was to relieve this distress that an operation was determined on. Perforation was made between the seventh and eighth ribs posteriorly, and about two quarts of pus, and a great quantity of air was discharged. The relief experienced was instantaneous. At the end of a week the cavity having again filled up the operation was repeated. Death took place in two weeks.

Dr. Rochester considered the mode of operation of great importance. The use of Dr. Wyman's instrument afforded a safe means of emptying the cavity without admitting external air, whereas when the ordinary canula was used this could not be avoided, and the result was the discharges became very offensive, and the danger to the patient increased.

*Dr. Miner* remarked upon the great interest of the case reported by Dr. Rochester, and the highly gratifying results of the case; partial recovery even, being a termination which must be regarded as remarkably favorable. Thought that the pump mentioned by Dr. Rochester might have advantages in operating, but doubted if the exclusion of external air was one of them. Must confess to a sympathy with the notion that exclusion of air from a pyogenic membrane was not so important as had generally been supposed. Could not see why external air should be worse than that admitted from within. Would like to inquire if Dr. R. ascribes any peculiar medicinal effects to camomile other than were possessed by the other bitter tonics? Had the opinion that camomile was a medicine of the feeblest properties, and like gum arabic and liquorice, incapable of doing any good or harm.

*Dr. Rochester* replied that Dr. Moore ascribed to the remedy in question almost specific properties in such cases. The idea was not original with Dr. M., but had been taken by him from some Medical Journal. Must confess himself rather skeptical on the subject, though he had tried it faithfully.

*Dr. Strong* thought there could not be much ground for doubt as to the propriety of the exclusion of air in such cases. Saw the operation performed some twenty-five years ago by his preceptor, in the old way, and he could never forget the extreme offensiveness of the discharges.

*Dr. Eastman* had employed chamomile for four or five years, in many cases, with good effect in diminishing the amount of pus. Generally used the fluid extract, and gave ℥ss three to five times daily. Thought the usual doses of ʒi to ʒij too small.

*Dr. Strong* thought chamomile could only be considered a good vegetable tonic and must deny it any specific properties.

*Dr. T. D. Strong* of Chautauqua county was introduced to the Association, and gave a highly interesting account of an epidemic of erysipelas which had been prevailing in that county. The substance of these remarks having already appeared in this Journal they are omitted here.

The diseases reported as prevailing were, infantile convulsions, pneumonia, mumps, erysipelas and roseola.

JOSEPH A. PETERS, Sec'y.

